# Effect of SGLT2 inhibitors on kidney function of type 2 diabetes patients during Ramadan: A systematic review

**DOI:** 10.5339/qmj.2024.26

**Published:** 2024-08-06

**Authors:** Muhammad Aamir Waheed, Salah Ali Saleh Diffala Suwileh, Khalid Rashid, Farrukh Ansar, Abdelnaser Elzouki

**Affiliations:** 1 College of Medicine, Hamad General Hospital, Qatar University, Doha, Qatar; 2 Hamad General Hospital, Doha, Qatar; 3 James Cook University Hospital, Middlesbrough, United Kingdom; 4 Department of Medicine, Quaid-e-Azam International Hospital, Islamabad, Pakistan; 5 Department of Medicine, Hamad General Hospital, Weill Cornell Medicine, Ar-Rayyan, Qatar

**Keywords:** SGLT2 inhibitors, kidney function, fasting, Ramadan, systematic review

## Abstract

**Background:**

: SGLT2 inhibitors are known for their osmotic diuretic effect, and their use by Muslim patients with type 2 diabetes during the fasting month of Ramadan may pose an increased risk of volume depletion, potentially impacting renal function.

**Methods:**

: We conducted a systematic review registered on PROSPERO (registration number CRD42020204582) of studies published between 2013 and January 2023, sourced from PubMed, EMBASE, and the Cochrane Central Register of Controlled Trials. The study selection criteria included controlled studies that reported the use of SGLT2 inhibitors (SGLT2i) by fasting adult type 2 diabetes patients and provided data on creatinine or estimated glomerular filtration rate (eGFR) as outcomes.

**Results:**

: Two prospective observational studies, encompassing a total of 359 participants, of which 197 utilized SGLT2 inhibitors, were identified. Our findings indicated that the use of SGLT2 inhibitors during Ramadan did not result in a significant alteration in eGFR. In one study by Hassanein et al., the mean changes in eGFR for the SGLT2i group, as compared to the non-SGLT2i group, were -1.2 ± 19.4 and 3.1 ± 14.8, respectively (*p* = 0.06). In a study by Shao et al., the least squares mean changes for eGFR in the SGLT2i group, compared to the non-SGLT2i group, were -6.0 ± 1.5 (95% CI, -8.9 to -3.1) and -4.2 ± 1.6 (95% CI, -7.3 to -1.1), respectively (*p* = 0.39).

**Conclusion:**

: Despite the limited number of observational studies available, our analysis suggests that the use of SGLT2 inhibitors by type 2 diabetes patients during Ramadan does not appear to significantly impact kidney function.

## INTRODUCTION

Sodium-glucose co-transporter-2 inhibitors (SGLT2i) are oral anti-hyperglycemic agents used to treat type 2 diabetes.^1^ The first agent was approved for clinical use in treating type 2 diabetes in 2013.^1^ They exert their effect by blocking glucose reabsorption in the proximal convoluted tubules of the kidney, which is the main site for glucose reabsorption, leading to osmotic diuresis.^1^ Since their approval, many published clinical trials have demonstrated their effectiveness and the beneficial protective clinical impact on cardiovascular and renal events.^2^ Initial studies have suggested an increase in the risk of acute kidney injury (AKI), which led the Food and Drug Administration to issue a warning regarding this risk.^3,4^ However, subsequent studies did not show an increased risk of AKI in SGLT2i users.^5,6^ In contrast to the findings of Perlman et al.,^3^ SGLT2i have been shown to decrease the incidence of AKI in a systematic review and meta-analysis that included 112 randomized controlled trials and 5 observational studies.^7^ At present, SGLT2i are the recommended drugs to augment standard care for type 2 diabetes patients with chronic kidney disease (CKD). This is attributed to their positive impact on kidney function.^8,9^

Ramadan is considered one of the holy months in the Islamic calendar for Muslims around the world. During Ramadan, all adult Muslims are required to abstain from taking food and drink for an average of 15 h a day for the duration of the full lunar month.^10^ The total hourly daily duration of fast depends on the geographic location. It is estimated that there are 148 million Muslims who live with diabetes globally.^11^ Fasting Ramadan may predispose patients to dehydration, which can be aggravated by the hot climate in tropical and subtropical areas where the majority of Muslims live.^12^ Although this concern for dehydration and volume depletion because of the use of SLT2 inhibitors during Ramadan is theoretic, it still raises concerns for the physicians caring for type 2 diabetes patients observing Ramadan based on a clinical survey result.^13^ In this survey, 136 physicians were asked about whether they consider SGLT2i safe and would prescribe them during Ramadan. Among the participants, 13.2% of them considered them as not safe, while 70.6% answered that they might be safe but should be discontinued in selected patients who might be at increased risk of adverse effects.^14^ To date, there are few small studies that evaluated the use of SGLT2i during Ramadan.^14,15,16^ These studies have addressed the risks of hypoglycemia, ketonemia, dehydration, and renal function.^15^ Some of these studies were examined in a systematic review to evaluate the efficacy and safety of oral hypoglycemic agents in diabetic patients during Ramadan, including SGLT2 inhibitors.^17^ However, the impact of SGLT2i on kidney function was not fully addressed in that review. The International Diabetes Federation (IDF) and the Diabetes and Ramadan (DAR) International Alliance have published practical guidelines on the management of diabetes during Ramadan in 2021.^18^ It was recommended that these drugs be initiated 2 weeks to a month before Ramadan to encourage hydration, and the impact on renal function should be considered. It was advised to half the dose of SGLT2i if the initiation of these medicines was intended for renal protection.

With the limited number of previous studies, small study samples, and the absence of any randomized controlled trial or systematic review to specifically address the real effect of SGLT2i on the kidney function of diabetic patients during Ramadan, we decided to conduct this systematic review to examine the available evidence so far to shed light on the renal safety of SGTLT2i during Ramadan, especially with the increasing number of patients using this class of medicine recently. Our goal was to examine whether Muslim patients with type 2 diabetes using SGLT2 inhibitors during the fasting month of Ramadan face an increased risk of volume depletion and assess its impact on renal function, focusing on safety concerns.

## METHODS

### Research Question Addressed

This systematic review was aimed at assessing the impact of Ramadan fasting on the estimated glomerular filtration rate (eGFR) in type 2 diabetes patients using sodium-glucose cotransporter 2 inhibitors (SGLT2i). This systematic review is registered on PROSPERO (registration number CRD42020204582).

### Data Sources and Searches

**Figure 1.**
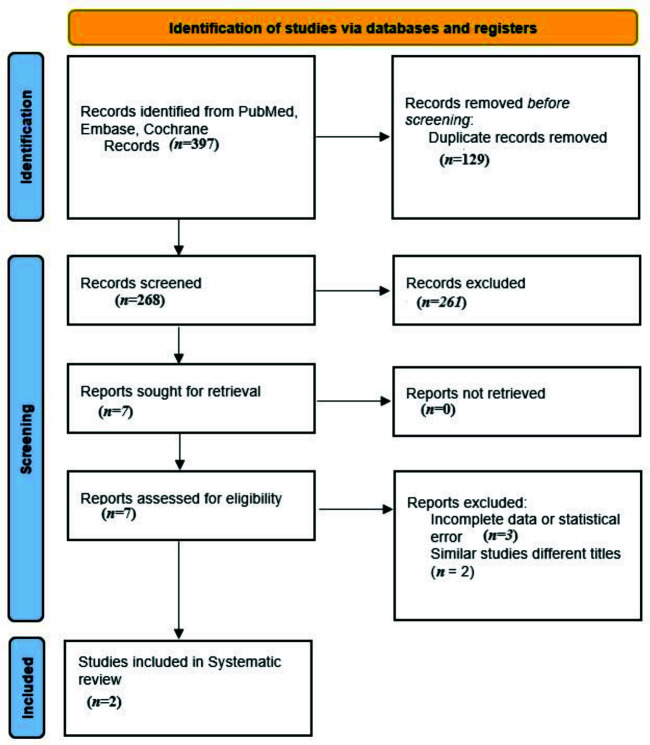
PRISMA flow diagram.

A thorough literature search was conducted from 2013 to September 18, 2022, with 2013 chosen as the starting point due to the FDA approval of the first SGLT2i. The search spanned major databases, including PubMed, Embase, and the Cochrane Central Register of Controlled Trials. Keywords encompassing themes such as “diabetes,” “SGLT2i,” “fasting,” “eGFR,” and “renal function” were combined using Boolean operators (AND and OR). Medical Subject Headings (MeSH) terms were intentionally omitted to maximize inclusiveness. Additionally, reference lists of retrieved studies were scrutinized to identify potentially missed research. As shown in Figure 1, 397 studies were identified in the initial search from PubMed, EMBASE, and Cochrane Central after removing the duplicates based on title screening. Out of these 397 studies, seven were selected for full text screening after applying the eligibility criteria in this review. From these seven studies, only two were included in the review, whereas the others have been excluded due to incomplete statistical data, non-extractable data, and studies conducted under different titles with similar objectives (Figure 1).

### Inclusion and Exclusion Criteria

The selection process for the studies in this review adhered to specific eligibility criteria. Inclusion criteria encompassed randomized controlled trials or observational studies involving adults aged 18 years and above with type 2 diabetes. These studies evaluated the use of sodium-glucose cotransporter 2 inhibitors (SGLT2i) during Ramadan and reported either creatinine or eGFR as renal endpoints. Furthermore, only studies published in the English literature from 2013 onward were considered. Conversely, exclusion criteria encompassed case reports, case series, case-control studies, and cross-sectional studies, along with studies lacking a control group.

To ensure a robust and unbiased selection process, two reviewers, namely, SS and MM, independently conducted the initial screening of the title and abstract for the studies retrieved from the initial search, following the established eligibility criteria and after the removal of duplicate records. Subsequently, a comprehensive full-text review of the selected studies was conducted for data extraction. In cases of any disagreements that arose between the two initial reviewers during the title and abstract screening, a constructive discussion was initiated to resolve the discrepancies. If a consensus could not be reached, a third reviewer, BM, was consulted to make a final decision, ensuring the integrity of the review process.

### Data Extraction and Quality Assessment

The relevant data that were extracted from the involved studies were:

1. Baseline characteristics of the study participants include age, sex, HbA1c, diabetes mellitus duration, systolic and diastolic blood pressure (BP), eGFR before enrollment, percent of participants using diuretics, and percentage of participants with CKD.
2. The study description includes the study design, authors, year published, and sample size.
3. The intervention used in all studies included drugs and their dosages, and the medicines used by the control group.
4. The outcome measurements are creatinine and/or eGFR measurements before and after fasting, systolic and diastolic blood pressure before and after Ramadan, and volume-related events like dehydration, postural hypotension, or dizziness.

The data were extracted by one reviewer (SS) and a second reviewer (BM) double-checked the extracted data. If any disagreement between the reviewers about data extraction occurs, then it was solved by discussion between them, with the final decision to be taken by the second reviewer (BM). We tried to contact the three corresponding authors through their emails for further information and explanations about their studies; however, none has responded back to us. Specifically, we requested further information about the justification for the use of median with standard deviation (SD) instead of mean with SD in the Abdulgadir et al.’s study.^19^ We also wanted to get further information about the mean change from baseline and the reason for many missed/unavailable observations for different variables for the participants in the Hassanein et al.’s study.^14^ For the Bashier et al.’s study,^20^ we requested the numeric data of the mean creatinine in both groups, which were not reported in the published article, although it was measured.

The Newcastle-Ottawa Scale (NOS) for assessing the quality of cohort studies was used in our review.^21^ Two reviewers (SS and BM) completed the risk of bias assessment task independently. The scale examines three main categories of a cohort study which are (A) selection of cohorts, (B) comparability of cohorts, and (C) outcome assessment. The selection categories comprise four items:

1. Representativeness of the exposed cohort
2. Selection of the non-exposed cohort
3. Ascertainment of exposure
4. Demonstration that outcome of interest was not present at start of study

Each of these items is star-rated based on certain criteria, and a maximum of one star is given for each item. In the comparability category, the comparability of cohorts based on the design or analysis is analyzed and star-rated based on defined criteria, and a maximum of two stars is granted for this category. The outcome category assesses three items: assessment of outcome, follow-up long enough for outcomes to occur, and adequacy of follow-up of cohorts. Again, each item is star-rated based on certain criteria, and a maximum of one star is given for each item. Based on the collected stars, the study will be rated as either having good, fair, or poor quality.

### Data Analysis

We performed a narrative systematic analysis of small studies using descriptive statistics and summaries, as a meta-analysis was not feasible due to differences in statistical methods. Table 1 presents baseline characteristics, with eGFR as the primary renal outcome measurement. The studies utilized different statistical analyses, preventing meta-analysis. Variability was represented by the SD for eGFR and the standard error (SE) for the least squares (LS) mean. The measure of variability for the eGFR and outcome variable mean changes was represented by the SD, and for the LS mean, the SE was used. We employed a two-sample *t*-test for comparing mean changes, with statistical significance set at a *p*-value < 0.05. Minitab 20 Statistical Software, Microsoft Windows 10, State College, PA, USA: Minitab, Inc., was used for statistical analysis.

### Ethical Aspects

This systematic review relies solely on secondary data from published sources, involving no primary data collection, experimentation, or intervention with human subjects. As our research is based on existing data, ethical approval was not necessary. We have strictly followed ethical guidelines and best practices in compiling and analyzing publicly available, previously published data to maintain the highest level of integrity in our review process.

## RESULTS

### Description of Studies

Three hundred and ninety-seven studies were identified in the initial search from PubMed, EMBASE, and Cochrane Central after removing the duplicates based on title screening. Out of these 397 studies, seven were selected for full text screening after applying the eligibility criteria in this review. From these seven studies, only two were included in the review, whereas the others have been excluded due to a variety of reasons. Two studies were excluded after they were found to be duplicates of already reviewed studies with a difference in titles in one pair^2,14^ and the other pair^20,23^ was an abstract and a published paper of the same study with two different authors for each. One study had no data for renal outcome measurement, one study had no numerical data for creatinine, and one study had a statistical error in outcome calculation in which the study outcome was measured as median with SD instead of mean with SD.^19^ The two studies were prospective observational and had a good quality design as assessed by NOS. The renal outcomes were secondary endpoints in both studies, and the measurements used in these studies were eGFR before and after fasting and creatinine in the Hassanein et al.’s study only. The SGLT2i used were canagliflozin in both and empagliflozin only in the Shao et al.’s study. In the Shao et al.’s study, 42% of the treatment group was not on full dose canagliflozin and dapagliflozin, while in the Hassanein et al.’s study, 83% of the treatment group was not on full dose canagliflozin. The total population of both studies was 359, out of which 197 used SGLT2i, while 162 used other medicines, including insulins and dipeptidyl peptidase-4 inhibitors. Participants were followed up for different durations in the two studies. In the Shao et al.’s study, the follow-up for renal outcome measurements occurred at least 1 week after starting the fast, while in the Hassanein et al.’s study, the follow-up measurements were taken between 4 and 8 weeks of fasting. In the Shao et al.’s study, 94% and 88% of the treatment and control groups successfully completed the study, respectively. In the Hassanein et al.’s study, less than 4% and 11% of the treatment and control groups missed more than 4 days of fasting, respectively.

### Description of Study Participants

The mean ages of participants ranged between 50 and 55 years. Of them, 54–62% were male. The mean pre fast eGFR of participants ranged from 84.4 to 91.2 ml/min/1.73 m^2^. Both studies had patients with CKD stages 1–3 (in the Hassanein et al.’s study, participants with CKD stage 3 and above were excluded); however, the percentage of those patients was not reported. Only six participants were on diuretics in the Shao et al.’s study. Further details and a more detailed description of baseline characteristics are presented in Table 1.

### Renal Outcomes

Treatment with SGLT2i during Ramadan did not significantly alter the eGFR values after starting the fast. Table 2 shows the non-significant changes in the mean and least squares mean after fasting in the Hassanein et al.’s and Shao et al.’s studies, respectively. However, there was a higher amount of missing eGFR data from the non-SGLT2 inhibitors group than in the SGLT2i group, 40% vs. 24% of both study groups, respectively, in the Hassanein et al.’s study.

### BP and Volume-related Events

The systolic and diastolic blood pressures after fasting were lower in the SGLT2i users in the two studies (Table 3). There was no difference in the number of postural hypotension events (defined as a drop in systolic BP ≥ 20 mmHg) among SGLT2i and non-SGLT2i users in the Shao et al.’s study (11/29 (37.9%) and 10/33 (30.3%), respectively, *p* = 0.527). However, there were more volume-related events (dehydration, postural dizziness, and hypovolemia) in SGLT2i than non-SGLT2i users in the Hassanein et al.’s study: 15/162 (9.3%) vs. 6/159 (3.8%), respectively.

## DISCUSSION

In conducting this review, we aimed to provide a comprehensive evaluation of the impact of SGLT2 inhibitors on kidney function in individuals with type 2 diabetes during the Ramadan fasting period. Notably, this systematic review stands out as the first of its kind, tailored specifically to assess the nuanced effects of SGLT2 inhibitors on renal function during Ramadan. Our findings indicate that the use of SGLT2 inhibitors among type 2 diabetes patients adhering to Ramadan practices did not yield a significant alteration in kidney function, as evidenced by eGFR levels. While the study by Hassanein et al. highlighted a greater incidence of volume-related events among SGLT2 inhibitor users, likely attributable to the osmotic diuresis associated with this medication, it is crucial to note that these events were not severe and did not necessitate the cessation of Ramadan fasting in that particular study. Furthermore, our review aligns with previous research, emphasizing the consistent association between SGLT2 inhibitor use and a notable reduction in both systolic and diastolic blood pressure.^24^ Our research findings unfold through several layers, offering a nuanced perspective. First, it is essential to consider the timeline of SGLT2i usage by our participants. Many had been on these medications for several months before joining the study. Notably, previous research suggests that the diuretic effects of these drugs tend to be transient, predominantly manifesting in the initial stages of treatment.^25^ Moreover, the timing of eGFR or creatinine measurement for some participants in the Shao et al.’s study occurred as early as 1 week into fasting. This early measurement poses a challenge in capturing the comprehensive impact of SGLT2i on kidney function, especially considering the prolonged fasting period of 29–30 days. Second, it is crucial to acknowledge the composition of our study population. High-risk groups, such as the elderly and individuals with CKD stage 3 or above, were either underrepresented or excluded from our research. This decision aligns, in part, with the guidelines outlined in the Diabetes and Ramadan practical guidelines by the IDF, in collaboration with the DAR International Alliance.^11^ These guidelines advise against the use of SGLT2i during Ramadan for elderly patients, shedding light on the careful considerations that shape the demographic composition of our study. Regarding patients with CKD, this could be justified by the fact that patients with CKD stage 3 and above are generally not advised to fast Ramadan.^11^ Nevertheless, many patients decide to fast during the holy month of Ramadan despite these advices for religious commitments. Third, we observed the limited use of diuretics involved which can add to the diuretic effect of SGLT2i and lead to volume depletion, which might worsen kidney function. It is assumed this is because the IDF and DAR guidelines do not recommend using SGLT2i with diuretics and the theoretical concern for dehydration and volume depletion.^11^ Prior research has demonstrated the renal protective effect of SGLT2i in type 2 diabetes patients in general.^26,27,22^ Among type 2 diabetes patients with normal kidney function who fast Ramadan, the change in creatinine after fasting was significantly increased, however, within the normal range.^28^ The research investigating the use of SGLT2i among type 2 diabetes patients is limited to a few studies; not all of them have evaluated the impact on kidney function. A prospective study with 417 participants in the United Arab Emirates using SGLT2i during Ramadan evaluated the change in creatinine level pre- and post-Ramadan and found no significant changes in creatinine levels after Ramadan.^20^ This adds to the findings of our study, which suggest the safety of SGLT2i usage during Ramadan.

There are other potential limitations to our review that limit the generalizability of this review. First, the involved studies are observational in nature and are only two in number, and the total number of participants is little; 389 participants in total, 197 only used SGLT2i. This is due to the rarity of research being done in these specific fields. Second, the kidney function surrogate endpoints used for the primary outcomes differed between the studies. Besides that, there are many eGFR equations for calculation, which can yield different results by using different equations. Also, the use of eGFR as a surrogate endpoint in clinical trials evaluating kidney disease over a short period of time might not be the perfect biomarker to be used. Third, there was significant missing data on follow-up in the Hassanein et al.’s study, which could have influenced the outcome results. Finally, there is limited representativeness of the elderly, people with CKD, and those on diuretics. Nevertheless, there are some strength points in this review. The included studies are graded as having good quality by applying the Newcastle-Ottawa Scale for assessing the risk of bias for cohort studies. The primary outcome effect trends were consistent among all studies, and there were no large differences in the baseline characteristics of the study participants, which included people from different geographical areas with different ethnicities (Table 1).

## CONCLUSION

The use of SGLT2i by type 2 diabetes patients during Ramadan may be safe and may not alter kidney function. However, as this review included only two studies and along with the above-mentioned limitations, future research is warranted to further evaluate the clinical use of SGLT2i by type 2 diabetes patients during Ramadan, with particular attention to the elderly patients, those with CKD, and those on diuretics.

## Declarations

### Acknowledgments

None.

### Conflict of interest

The authors declare that they have no conflict of interests.

## Figures and Tables

**Table 1.** Descriptive summary of native speakers worldwide and the number of publications on PubMed (From inception until December 2023).

Studies characteristics	Shao Y 2018		Hassanein M 2017	
	SGLT2	Non-SGLT2	SGLT2	Non-SGLT2
Study size, *n*	35	33	162	159
Age (years), mean (SD)	50 (11)	55 (11)	52 (8)	54 (7)
Sex (males %)	54%	55%	62%	55%
*HbA*1c, %, mean (SD)	9.3 (1.9)	8.7 (1.6)	7.3 (0.8)	7.2 (0.8)
*HbA*1c, mmol/mol, mean (SD)	78 (18.6)	72 (15.3)	56 (6.5)	55 (6.5)
Diabetes mellitus duration, years, mean (SD)	12 (6)	16 (8)	7 (6)	8 (6)
SBP, mmHg, mean (SD)	138 (20)	143 (20)	129 (12)	130 (12)
DBP, mmHg, mean (SD)	79 (9)	82 (11)	78 (7)	77 (8)
eGFR, ml/min/1.73 m^2^, mean (SD)	91.2 (21.8)	84.9 (23.7)	89.9 (19.6)	88.7 (17.6)
On diuretics, *n* (%)	2 (5.7)	4 (12.1)	N/A	N/A
CKD stage	1-3		1-2	

CKD: chronic kidney disease, DBP: diastolic blood pressure, eGFR: estimated glomerular filtration rate, SBP: systolic blood pressure, SD: standard deviation.

**Table 2.** Descriptive summary of native speakers worldwide and the number of publications on PubMed (From inception until December 2023).

		SGLT2i	Non-SGLT2i	*p*-Value
Hassanein M 2017	Before fasting eGFR, mean (SD)	*n* = 162 89.9 (19.6)	*n* = 159 88.7 (17.6)	
	After fasting eGFR, mean (SD)	*n* = 123 88.4 (21.6)	*n* = 96 89.9 (19.9)	
	Mean change from baseline	*n* = 123 −1.2 (19.4)	*n* = 96 3.1 (14.8)	0.06
Shao Y 2018 eGFR, LS mean change (±SE)		−6.0 (± 1.5) (95% CI −8.9 to −3.1)	−4.2 (± 1.6) (95% CI −7.3 to −1.1)	0.39

eGFR is measured in ml/min/1.73 m^2^. CI: confidence interval, LS: least squares, SD: standard deviation, SE: standard error.

**Table 3.** Descriptive summary of native speakers worldwide and the number of publications on PubMed (From inception until December 2023).

		SGLT2i		Non-SGLT2i		
		Before Ramadan	After Ramadan	Before Ramadan	After Ramadan	*p*-Value
Hassanein M 2017	SBP, mmHg mean (SD)	129.0 (11.7) *n* = 162	127.9 (10.2) *n* = 155	129.8 (12.0) *n* = 158	130.7 (12.1) *n* = 150	
	Change from baseline	*n* = 155 −1.5 (12.9)		*n* = 149 1.2 (12.5)		
	DBP, mmHg mean (SD)	78.2 (6.7) *n* = 162	75.9 (7.3) *n* = 155	76.7 (8.0) *n* = 158	77.0 (7.8) *n* = 150	
	Change from baseline	*n* = 155 −2.9 (9.1)		*n* = 149 0.5(9.0)		
Shao Y 2018	SBP, mmHg mean (SD)	−8.1 (± 2.8) 95% −13.7 to −2.5		−10.4 (± 3.0) 95% CI −16.4 to −4.4		0.569
	DBP, mmHg mean (SD)	−3.7 (± 1.3) 95% −6.3 to −1.0		−3.5 (± 1.4) 95% −6.4 to −0.7		0.934

*n* = number of participants. CI: confidence interval, DBP: diastolic blood pressure, SBP: systolic blood pressure, SD: stanadrd deviation.
